# Application of Lanthanide Shift Reagent to the ^1^H-NMR Assignments of Acridone Alkaloids

**DOI:** 10.3390/molecules25225383

**Published:** 2020-11-17

**Authors:** Sio-Hong Lam, Hsin-Yi Hung, Ping-Chung Kuo, Daih-Huang Kuo, Fu-An Chen, Tian-Shung Wu

**Affiliations:** 1 School of Pharmacy, College of Medicine, National Cheng Kung University, Tainan 701, Taiwan; shlam@mail.ncku.edu.tw (S.-H.L.); z10308005@email.ncku.edu.tw (H.-Y.H.); z10502016@email.ncku.edu.tw (P.-C.K.); 2 Department of Pharmacy, College of Pharmacy and Health Care, Tajen University, Pingtung 907, Taiwan; dhkou@tajen.edu.tw (D.-H.K.); fachen@tajen.edu.tw (F.-A.C.)

**Keywords:** acridone alkaloid, lanthanide shift reagent, tris(dipivaloylmethanato)-europium(III)

## Abstract

This study investigates the application of the paramagnetic shift reagent tris(dipivaloylmethanato)-europium(III) in NMR spectral studies of permethoxyacridone alkaloids (**1**–**3**) and pyranoacridone alkaloids (**4**–**6**). The induced chemical shifts (∆δ) of all protons were observed for the same molecule, and were compared to deduce the positions resulting from the distance nearby the Eu(dpm)_3_. Assignment of the H-2, H-4 and H-8 of polysubstituted acridones could be distinguished based on the least-squares method of lanthanide-induced shifts plotted against the mole ratios of Eu(dpm)_3_ to the substrate. The developed method is not only potentially useful for determining the planar structures of polysubstituted compounds, such as acridones, anthraquinones, xanthones, flavonoids, and phenanthrenes, but also applicable for their stereochemistry.

## 1. Introduction

Acridone alkaloids have become an important topic of medicinal chemistry research in recent years, due to the broadband biological activities on the tricyclic acridine ring system for different design and discovery [1,2]. There are numerous pharmacological publications of the acridone alkaloids and their derivatives that have been reported previously, including anticancer [3,4], antivirus [5,6,7], antibacterial and antifungal [7], anticonvulsant [1], anti-acetylcholinesterase [8], etc. These features are attributed to the semiplanar heterocyclic structure, which interacts with different biomolecular targets proved by the docking studies [9]. Most acridone alkaloids were isolated from the relatively limited genus of Rutaceae, and have been found to be widely distributed across these species [10,11,12,13]. However, elucidation of the structures of new acridones with multiple substitutions is confused, in particular, the positions of H-2 and H-4. According to the literature survey, several structures of acridone alkaloid derivatives were assigned ambiguously based merely on ^1^H-NMR in the past [14,15]. The major problems of these compounds were polyoxygenated substitutions on the ring system leading to only one or two proton signals for aromatic substructure elucidation. Especially singlet proton signals at the C-2 or C-4 position of acridones are easily determined mistakenly and usually need chemical reaction for further construction of complete structures [16]. In our previous article, we have compiled the ^13^C NMR signals of the acridone alkaloids as an important reference for the determining substituted positions [17]. Herein, we wish to develop a method for solving such structural problems of acridones by using a shift reagent to establish a feasible technique for NMR determination of acridones and related compounds.

The Lanthanide shift reagents (LSR) introduced by Hinckley in 1969 are paramagnetic compounds that can induce paramagnetic shifts on the adjacent nuclear spins of the molecular system with which it interacts [18]. These reagents, such as tris(dipivaloylmethanato)-europium(III) [Eu(dpm)_3_], are useful for the structural analysis and stereochemical elucidation of lone pair containing molecules by NMR spectroscopy [19,20]. Lanthanides (elements 57 to 71) contain 4f electrons, usually do not participate in chemical bonding, and can provide greater paramagnetic contributions [18]. Magnetic interactions accompanying the association induce shifts in NMR spectra of the organic substrates, and the chemical shift difference is depending on the relative substrate/shift reagent concentration [20]. The higher the concentration of the shift reagent, the more significant the difference in chemical shift between the enantiomers. Additionally, the interaction of neighboring groups can be exploited to obtain structural information by using lanthanide shift reagents [18]. Lanthanide complexes that bind to organic molecules can thereby spread out proton resonances to simplify their analysis.

In this ^1^H NMR study related to naturally occurring compounds, we investigated the Eu(dpm)_3_-induced shift effects on the methyl proton resonances of several acridone alkaloids. Previous studies demonstrated that Eu(dpm)_3_ in protic or phenolic compounds was unstable and easily degraded. Hence, the phenolic functionalities of acridone alkaloids can be inactivated by conversion to the corresponding methoxy groups for further shift reagent application, and then the spectral interpretation is simplified. This extrapolation method of Eu(dpm)_3_-induced ^1^H NMR spectra have been applied to establish the unambiguous assignments.

## 2. Results and Discussion

**Figure 1:**
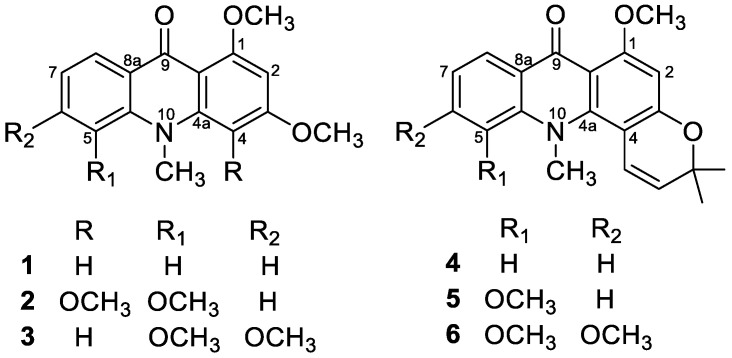
Structures of acridone alkaloids **1**–**6.**

**Figure 2:**
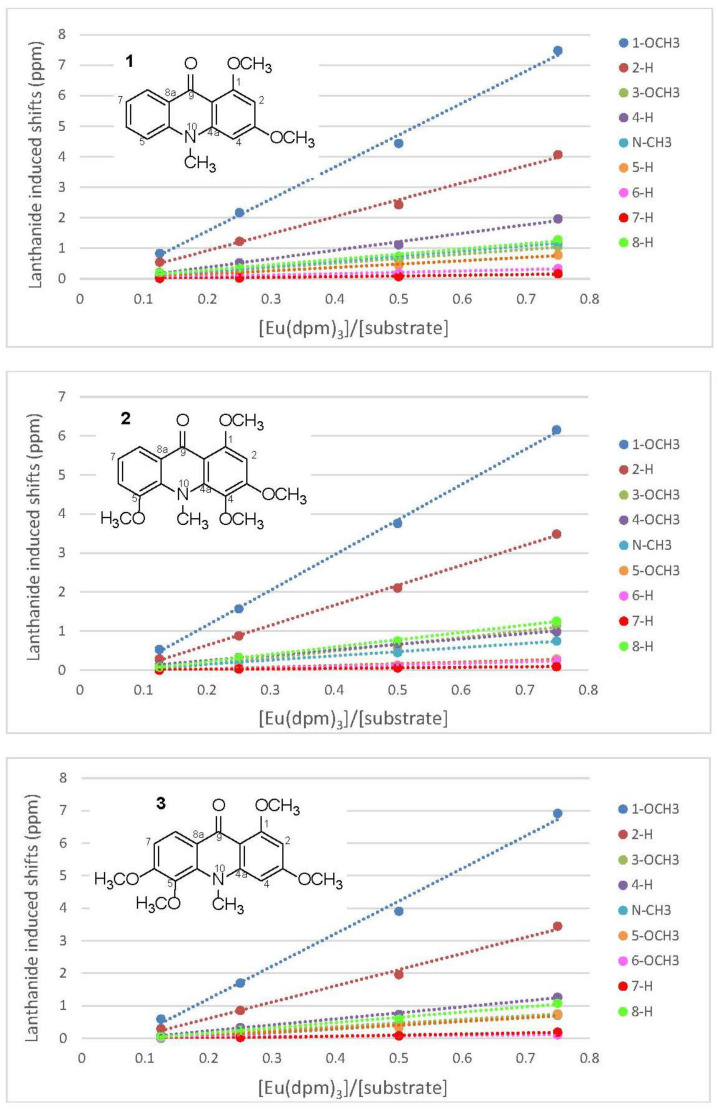
Lanthanide induced shifts of some signals of permethoxyacridones (**1**–**3**), plotted against the mole ratios of Eu(dpm)_3_ to the substrate.

**Figure 3:**
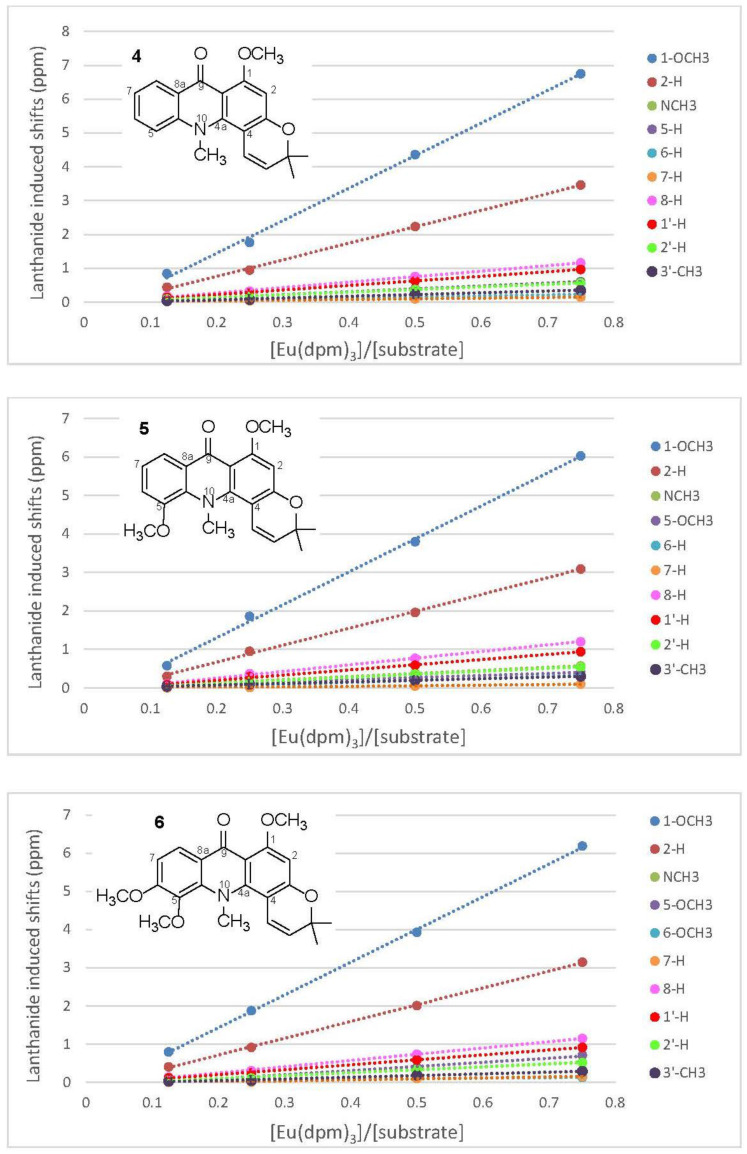
Lanthanide induced shifts of some signals of pyranoacridones (**4**–**6**), plotted against the mole ratios of Eu(dpm)_3_ to the substrate.

The phenolic acridone alkaloids, 1,3-dihydroxyacridone (**1a**), citrusinine-I (**2a**), citpressine-II (**3a**), noracronycine (**4a**), 5-hydroxynoracronycine (**5a**), and citracridone-II (**6a**) [21,22,23], were permethylated in acetone using anhydrous potassium carbonate and methyl iodide to obtain acridone alkaloids **1**–**6**, respectively (Figure 1). The intramolecular hydrogen bond between the C-1 hydroxyl group and the C-9 ketone group creates a force interaction, resulting in a prolonged reaction time. To increase the yield and reduce the reaction time, a few drops of DMSO were added to accelerate the reaction successfully. The ^1^H NMR spectra of 1,3-dimethoxy-N-methylacridone (**1**), 1,5-O,O-dimethylcitrusinine-I (**2**), 1-O-methylcitpressine-II (**3**), acronycine (**4**), 5-methoxyacronycine (**5**) and 1-O-methylcitracridone-II (**6**) were measured in CDCl_3_. Lanthanide-induced shift studies with Eu(dpm)_3_ were carried out with acridones **1**–**6**. Tables S1–S6 summarize the chemical shifts (in ppm) observed before adding Eu(dpm)_3_, and the shifts induced by different molar ratios of Eu(dpm)_3_/substrate. On successive addition of lanthanide shift reagent, all of the protons were deshielded without notable line broadening effects. In the presence of LSR, the signal assignments were almost entirely based on the generalization that a proton close to the oxygen-containing site of the Eu^3+^ was moved more significantly than the protons further far away. Plots of the chemical shifts of all the protons of acridone alkaloids (**1**–**6**) vs. the molar ratios of added to the substrate all gave a straight line within the molar ratio from 0.125–0.75, as shown in Figure 2 and Figure 3.

1,3-Dimethoxy-N-methylacridine (**1**) and acronycine (**4**) were selected as representative compounds because they are unsubstituted at the C-2 and C-4 positions. We discuss the different effects of LSR in inducing chemical shifts difference into two classes of compounds, i.e., permethoxyacridone (**1**–**3**) and pyranoacridone (**4**–**6**).

### 2.1. Eu(dpm)_3_-Induced ^1^H-Chemical Shifts Difference of Permethoxyacridone (**1**–**3**)

The ^1^H NMR spectrum of 1,3-dimethoxy-N-methylacridine (**1**) showed the existence of two methoxy (δ 3.90, 3.97) and N-methyl groups (δ 3.69). In aromatic region, two relatively higher field signals at δ 6.14 (1H, d, *J* = 2.0 Hz, H-2) and 6.31 (1H, d, *J* = 2.0 Hz, H-4) indicated a meta coupling aromatic system. Additional four protons at δ 7.18 (1H, d, *J* = 8.0 Hz, H-7), 7.29 (1H, dd, *J* = 8.0, 2.0 Hz, H-5), 7.55 (1H, dt, *J* = 8.0, 2.0 Hz, H-6), 8.44 (1H, dd, *J* = 8.0, 2.0 Hz, H-8) displayed mutual coupling by adjacent protons. After adding the shift reagent, the chemical shift of OCH_3_-1 (δ 3.97) changed most obviously. The differences in the chemical shift of OCH_3_-1 was increased by 0.83, 2.17, 4.44, and 7.48 ppm at the Eu(dpm)_3_/substrate molar ratios of 0.125, 0.25, 0.5, and 0.75, respectively (see Table S1). The slope of the OCH_3_-1 regression line was 10.5 when the coefficient of determination (r^2^) equal to 0.996. For H-2 (δ 6.14), the slope of the regression line was 5.55. When the molar ratios were 0.125, 0.25, 0.5, and 0.75, the chemical shifts increased by 0.55, 1.23, 2.43, and 4.07 ppm, respectively (see Table S1). The chemical shifts of the other two aromatic protons at δ 8.44 (H-8) and δ 6.31 (H-4) had a moderate increasing trend after the shift reagent was added in different molar ratios. The slope of their regression line was between 1.7 to 2.8. The chemical shifts of the remaining proton signals were less changed after adding different molar ratios of LSR. Similar phenomena also appeared in 1,5-O,O-dimethylcitrusinine-I (**2**), and 1-O-methylcitpressine-II (**3**). The regression line slopes of OCH_3_-1 (δ 3.98), H-2 (δ 6.36), and H-8 (δ 7.86) of compound **2** were 9.00, 5.11, and 1.87, respectively (see Table S2). The chemical shift enhancement of OCH_3_-4 (δ 3.78) of compound **2** was lower than that of OCH_3_-3 (δ 3.98). Compound **3** possessed tetramethoxy substituents at C-1, C-3, C-5, and C-6. The regression line slopes of OCH_3_-1 (δ 3.98) and H-2 (δ 6.25) were also greater than other signals in **3** (see Table S3). Among compounds **1**–**3**, the chemical shift inducing of N-CH_3_ caused by adding LSH was less than those of the above signals OCH_3_-1, H-2, and H-8. All the data were summarized in Tables S1–S3 and shown as the plot in Figure 2 by the least-squares method.

### 2.2. Eu(dpm)_3_-Induced ^1^H Chemical Shifts Difference of Pyranoacridone (**4**–**6**)

The structure of acronycine (**4**) possessed an acridone basic skeleton with a dimethylpyran ring moiety. In its ^1^H-NMR spectrum, two methyl signals bearing heteratom at δ 3.79 (3H, s) and 3.92 (3H, s) were assigned as N-methyl and methoxy group, respectively. In the aromatic region, four mutually coupling signals located at δ 7.17 (1H, t, *J* = 8.0 Hz), 7.28 (1H, dd, *J* = 8.0, 2.0 Hz), 7.56 (1H, dt, *J* = 8.0, 2.0 Hz), 8.32 (1H, dd, *J* = 8.0, 2.0 Hz) were attributed to H-7, H-5, H-6 and H-8, respectively. The signals at δ 1.56 (6H, s, 2 × CH_3_), 5.46 (1H, d, *J* = 10.0 Hz, H-2’), and 6.49 (1H, d, *J* = 10.0 Hz, H-1’) indicated the presence of 2,2-dimethylpyrano moiety. The remaining singlet proton signal at δ 6.27 (1H, s, H-2) represented a penta-substituted aromatic ring system. The addition of LSR resulted in a large change in the chemical shifts of OCH_3_-1 (δ 3.92), H-2 (δ 6.27) and H-8 (δ 8.32). The regression line slopes of OCH_3_-1, H-2, and H-8 of **4** were 9.62, 4.89, and 1.61, respectively (see Table S4). In addition, the same experiments were performed on 5-methoxyacroycine (**5**) and 1-*O*-methylcitracridone (**6**), and the acquired results were similar to those of **4**. All the original data were provided in Tables S4–S6 and shown as plots in Figure 3 by the least-squares method.

**Figure 4:**
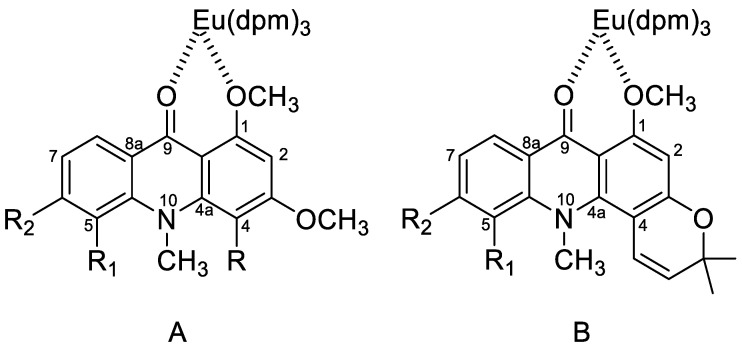
Lanthanide permethoxyacridones complexes and pyranoacridones complexes.

According to the experimental results discussed above, after adding different concentrations of the LSR, the order of the chemical shift of proton signals was OCH_3_-1 > H-2 > H-4/H-8. Regarding the structures of acridone alkaloids, the europium of the LSR used this time was generally considered to coordinate with the carbonyl group (C-9). However, the increment in the chemical shift of the H-2 signal was greater than that of the H-8 signal. These results indicated that both the carbonyl oxygen atom and one of the neighboring methoxy-oxygens were also involved in the coordination of europium, which provided greater paramagnetic contributions of the neighbor H-2 signal (Figure 4). Consequently, after using LSR, the signal of H-2 would be significantly shifted, and whether there was a substituent at position 2 or 4 can be clearly identified. At the same time, the binding direction of the pyran ring can also be determined accordingly.

The present method is potentially useful for determining the structures of polysubstitution compounds, such as acridones, anthraquinones, xanthones, flavonoids, and phenanthrenes, including their stereochemistry. The induced differences in the ^1^H-NMR chemical shifts between all protons of the acridones were extensively enhanced by the use of varieties concentrations of Eu(dpm)_3_. The effect of adding different concentrations of the shift reagent on proton resonances obviously was depended on the spatial relationship of functional groups, and this made it easier to differentiate the position of each proton. The employed methodology was sensitive to NMR signal assignments and did not produce numerical solutions with meaningless chemical interpretation. Although high magnetic fields NMR and 2D techniques are convenient and commonly used for elucidating chemical structures in recent years, it was still very difficult to determine the structures of some polysubstituted compounds, due to their ambiguities. In this case, the derivatization of the target compound is generally required to provide more information and obtain the exact structure [24]. Compared to the chemical modification, the shift reagent method described herein will be a more feasible alternative to determine the accurate structure conveniently. Since this method will not cause compound degradation and by-products formation, it can provide more definitive results than the couple of possibilities constructed with the traditional NMR spectral elucidation techniques.

## 3. Experimental Section

### 3.1. General

Six acridone alkaloids, including 1,3-dihydroxyacridone (**1a**), citrusinine-I (**2a**), citpressine-II (**3a**), noracronycine (**4a**), 5-hydroxynoracronycine (**5a**), and citracridone-II (**6a**), were purified from Rutaceae plant materials in our laboratory, and their structures were identified by comparison of physical and spectroscopic data with those reported in the literature [21,22,23]. All chemical reagent (CAS grade) were purchased from Wako Chemicals (Japan). IR and UV were determined on a JASCO IRA-I Infrared Spectrometer and a JASCO UVIDEC-I Spectrophotometer, respectively. 1D and 2D NMR spectra were recorded in CDCl3 with TMS as internal standard on a PS-100 (JEOL) spectrometer. Mass spectra were measured on a HITACHI M-52 mass spectrometer.

### 3.2. Permethylation of Acridone Alkaloids

50 mg of each phenolic acridone alkaloids, including 1,3-dihydroxyacridone (**1a**), citrusinine-I (**2a**), citpressine-II (**3a**), noracronycine (**4a**), 5-hydroxynoracronycine (**5a**), and citracridone-II (**6a**), was dissolved in 50 mL acetone. Anhydrous potassium carbonate (2 g) and methyl iodide (3 mL) with 0.2 mL dimethylsulfoxide were added and refluxed for 6 h. The crude product was filtered and recrystallized from diethyl ether to yield **1**–**6**, respectively.

#### 3.2.1. 1,3-Dimethoxy-N-methylacridone (**1**)

Pale yellow needles; m.p. 156–158 °C; UV λ_max_ 212, 225, 261, 267, 292, 315 (sh), 382 nm; IR (KBr) ν_max_ 1600, 1560, 1500 cm^–1^; MS *m/z* 269 (M^+^, 100%); ^1^H NMR (CDCl_3_, 100 MHz) *δ*_H_ 3.69 (3H, s, *N*-CH_3_), 3.90 (3H, s, 3-OCH_3_), 3.97 (3H, s, 1-OCH_3_), 6.14 (1H, d, *J* = 2.0 Hz, H-2), 6.31 (1H, d, *J* = 2.0 Hz, H-4), 7.18 (1H, t, *J* = 8.0 Hz, H-7), 7.29 (1H, dd, *J* = 8.0, 2.0 Hz, H-5), 7.55 (1H, td, *J* = 8.0, 2.0 Hz, H-6), 8.44 (1H, dd, *J* = 8.0, 2.0 Hz, H-8).

#### 3.2.2. 1,5-O,O-Dimethylcitrusinine-I (**2**)

Colorless needles; m.p. 160–162 °C; UV λ_max_ 207, 222, 261, 312, 395 nm; IR (KBr) ν_max_ 1625, 1595, 1580, 1560, 1505 cm^–1^; MS *m/z* 329 (M^+^), 314 (100%), 299, 270; ^1^H NMR (CDCl_3_, 100 MHz) *δ*_H_ 3.63 (3H, s, *N*-CH_3_), 3.78 (3H, s, 4-OCH_3_), 3.98 (9H, s, 1,3,5-OCH_3_), 6.36 (1H, s, H-2), 7.10 (1H, dd, *J* = 7.0, 2.0 Hz, H-6), 7.15 (1H, t, *J* = 7.0 Hz, H-7), 7.86 (1H, dd, *J* = 7.0, 2.0 Hz, H-8).

#### 3.2.3. 1-O-Methylcitpressine-II (**3**)

Pale yellow needles; m.p. 121–122 °C; UV λ_max_ 215, 230 (sh), 267, 286 (sh), 321, 378 nm; IR (KBr) ν_max_ 1625, 1590, 1555, 1510 cm^–1^; MS *m/z* 329 (M^+^, 100%), 314, 312, 300, 298, 284, 270; ^1^H NMR (CDCl_3_, 100 MHz) *δ*_H_ 3.76 (3H, s, 5-OCH_3_), 3.89 (3H, s, 3-OCH_3_), 3.92 (3H, s, 6-OCH_3_), 3.97 (6H, s, *N*-CH_3_ and 1-OCH_3_), 6.25 (1H, d, *J* = 2.0 Hz, H-2), 6.37 (1H, d, *J* = 2.0 Hz, H-4), 6.88 (1H, d, *J* = 9.0 Hz, H-7), 8.14 (1H, d, *J* = 9.0 Hz, H-8).

#### 3.2.4. Acronycine (**4**)

Pale yellow needles; m.p. 175–176 °C; UV λ_max_ 224, 260 (sh), 281, 292, 307, 395 nm; IR (KBr) ν_max_ 1620, 1590, 1560 cm^–1^; MS *m/z* 321 (M^+^), 306 (100%), 292, 276, 261; ^1^H NMR (CDCl_3_, 100 MHz) *δ*_H_ 1.56 (6H, s, 2 × CH_3_), 3.79 (3H, s, *N*-CH_3_), 3.92 (3H, s, 1-OCH_3_), 5.46 (1H, d, *J* = 10.0 Hz, H-2′), 6.27 (1H, s, H-2), 6.49 (1H, d, *J* = 10.0 Hz, H-1′), 7.17 (1H, t, *J* = 8.0 Hz, H-7), 7.28 (1H, dd, *J* = 8.0, 2.0 Hz, H-5), 7.56 (1H, td, *J* = 7.0, 2.0 Hz, H-6), 8.32 (1H, dd, *J* = 7.0, 2.0 Hz, H-8).

#### 3.2.5. 5-Methoxyacronycine (**5**)

Pale yellow needles; m.p. 185–187 °C; UV λ_max_ 207, 232, 264, 280, 291 (sh), 325, 335 (sh), 395 nm; IR (KBr) ν_max_ 1630, 1620, 1595, 1575, 1560 cm^–1^; MS *m/z* 351 (M^+^), 336 (100%), 322, 306, 292; ^1^H NMR (CDCl_3_, 100 MHz) *δ*_H_ 1.53 (6H, s, 2 × CH_3_), 3.59 (3H, s, *N*-CH_3_), 3.94 (3H, s, 5-OCH_3_), 3.97 (3H, s, 1-OCH_3_), 5.54 (1H, d, *J* = 10.0 Hz, H-2′), 6.29 (1H, s, H-2), 6.66 (1H, d, *J* = 10.0 Hz, H-1′), 7.07 (1H, dd, *J* = 7.0, 2.0 Hz, H-6), 7.18 (1H, t, *J* = 7.0 Hz, H-7), 7.88 (1H, dd, *J* = 7.0, 2.0 Hz, H-8).

#### 3.2.6. 1-O-Methylcitracridone-II (**6**)

Pale yellow needles; m.p. 125–127 °C; UV λ_max_ 207, 225, 271, 291 (sh), 335, 386 nm; IR (KBr) ν_max_ 1630, 1620, 1595, 1570, 1500 cm^–1^; MS *m/z* 381 (M^+^), 366 (100%), 352, 336, 322; ^1^H NMR (CDCl_3_, 100 MHz) *δ*_H_ 1.53 (6H, s, 2 × CH_3_), 3.62 (3H, s, *N*-CH_3_), 3.89 (3H, s, 6-OCH_3_), 3.93 (3H, s, 5-OCH_3_), 3.95 (3H, s, 1-OCH_3_), 5.55 (1H, d, *J* = 10.0 Hz, H-2′), 6.28 (1H, s, H-2), 6.59 (1H, d, *J* = 10.0 Hz, H-1′), 6.88 (1H, d, *J* = 9.0 Hz, H-7), 8.01 (1H, d, *J* = 9.0 Hz, H-8).

### 3.3. Lanthanide Shift Reagent Preparation

Each permethylated acridone alkaloid (**1**–**6**, 0.05 mmole) was dissolved in 0.5 mL CDCl_3_ in an NMR tube. The ratio ratios of [Eu(dpm)_3_]/substrate was prepared into 0.125, 0.25, 0.5, and 0.75 for ^1^H NMR measurement.

## Supplementary Materials

The following are available online at Tables S1–S6: original chemical shifts data of compounds **1**–**6**.
