# Supplementary material for: Application of Lanthanide Shift Reagent to the 1H-NMR Assignments of Acridone Alkaloids
Source: Molecules. 2020 Nov 17;25(22):5383. doi: 10.3390/molecules25225383 (PMC7698746; doi:10.3390/molecules25225383)
Supplement: Supplementary file 1 [file molecules-25-05383-s001.pdf]

Supplementary materials

Application of Lanthanide Shift Reagent to the ^{1}H-NMR Assignments of Acridone Alkaloids

Sio-Hong Lam [1], Hsin-Yi Hung [1], Ping-Chung Kuo [1], Daih-Huang Kuo [2], Fu-An Chen [2] and Tian-Shung Wu [1,2,*]

[1] School of Pharmacy, College of Medicine, National Cheng Kung University, Tainan 701, Taiwan; shlam@mail.ncku.edu.tw (S.-H.L.); z10308005@email.ncku.edu.tw (H.-Y.H.); z10502016@email.ncku.edu.tw (P.-C.K.)

[2] Department of Pharmacy, College of Pharmacy and Health Care, Tajen University, Pingtung 907, Taiwan; dhkou@tajen.edu.tw (D.-H.K.); fachen@tajen.edu.tw (F.-A.C.)

* Correspondence: tswu@mail.ncku.edu.tw; Tel.: +886-6-2757575 (ext. 65333)

Received: 23 October 2020; Accepted: 16 November 2020; Published: 17 November 2020

Contents

Table S1. Chemical shifts (δ, ppm) and chemical shifts difference (Δδ, ppm) of compound **1** under different mole ratios of $Eu(dpm)_3$ to the substrate.

Table S2. Chemical shifts (δ, ppm) and chemical shifts difference (Δδ, ppm) of compound **2** under different mole ratios of $Eu(dpm)_3$ to the substrate.

Table S3. Chemical shifts (δ, ppm) and chemical shifts difference (Δδ, ppm) of compound **3** under different mole ratios of $Eu(dpm)_3$ to the substrate.

Table S4. Chemical shifts (δ, ppm) and chemical shifts difference (Δδ, ppm) of compound **4** under different mole ratios of $Eu(dpm)_3$ to the substrate.

Table S5. Chemical shifts (δ, ppm) and chemical shifts difference (Δδ, ppm) of compound **5** under different mole ratios of $Eu(dpm)_3$ to the substrate.

Table S6. Chemical shifts (δ, ppm) and chemical shifts difference (Δδ, ppm) of compound **6** under different mole ratios of $Eu(dpm)_3$ to the substrate.

Table S1. Chemical shifts (δ, ppm) and chemical shifts difference (Δδ, ppm) of compound **1** under different mole ratios of $Eu(dpm)_3$ to the substrate.

ratio / gp	+0	+0.125	+0.25	+0.5	+0.75	a	b	r^2	Lower 95%	Upper 95%
	δ	Δδ	Δδ	Δδ	Δδ					
1-OCH_3	3.97	0.83	2.17	4.44	7.48	10.4786	–0.5269	0.9959	8.4322	12.5251
2-H	6.14	0.55	1.23	2.43	4.07	5.5539	–0.1863	0.9949	4.3413	6.7665
3-OCH_3	3.90	0.12	0.30	0.62	1.06	1.4834	–0.0776	0.9946	1.1501	1.8167
4-H	6.31	0.21	0.53	1.11	1.97	2.7742	–0.1720	0.9910	1.9696	3.5789
N-CH_3	3.69	0.12	0.34	0.70	1.19	1.6827	–0.0961	0.9956	1.3415	2.0239
5-H	7.29	0.09	0.20	0.45	0.78	1.1010	–0.0673	0.9928	0.8164	1.3856
6-H	7.55	0.05	0.10	0.19	0.34	0.4556	–0.0151	0.9843	0.2806	0.6306
7-H	7.18	0.02	0.03	0.07	0.17	0.2373	–0.0239	0.9220	0.0273	0.4473
8-H	8.44	0.20	0.36	0.74	1.28	1.7220	–0.0546	0.9883	1.1528	2.2913

a = slope of the regression line. b = intercept. r^2 = coefficient of determination. Lower 95% confidence interval. Upper 95% confidence interval.

Table S2. Chemical shifts (δ, ppm) and chemical shifts difference (Δδ, ppm) of compound **2** under different mole ratios of $Eu(dpm)_3$ to the substrate.

ratio / gp	+0	+0.125	+0.25	+0.5	+0.75	a	b	r^2	Lower 95%	Upper 95%
	δ	Δδ	Δδ	Δδ	Δδ					
1-OCH_3	3.98	0.53	1.57	3.76	6.16	9.0061	–0.6537	0.9992	8.2143	9.7979
2-H	6.36	0.29	0.88	2.11	3.49	5.1132	–0.3847	0.9989	4.5882	5.6383
3-OCH_3	3.98	0.06	0.22	0.57	1.15	1.7247	–0.2007	0.9802	0.9799	2.4696
4-OCH_3	3.78	0.12	0.33	0.68	0.99	1.3831	–0.0319	0.9969	1.1500	1.6161
N-CH_3	3.63	0.08	0.21	0.45	0.75	1.0617	–0.0588	0.9973	0.8950	1.2284
5-OCH_3	3.98	0	0.06	0.13	0.29	0.4447	–0.0607	0.9699	0.2065	0.6830
6-H	7.10	0	0.03	0.11	0.25	0.3973	–0.0639	0.9707	0.1873	0.6073
7-H	7.15	0	0.03	0.06	0.09	0.1383	–0.0112	0.9797	0.0777	0.1989
8-H	7.86	0.08	0.32	0.75	1.26	1.8698	–0.1571	0.9986	1.6601	2.0795

a = slope of the regression line. b = intercept. r^2 = coefficient of determination. Lower 95% confidence interval. Upper 95% confidence interval

Table S3. Chemical shifts (δ, ppm) and chemical shifts difference (Δδ, ppm) of compound 3 under different mole ratios of Eu(dpm)3 to the substrate.

ratio / gp	+0	+0.125	+0.25	+0.5	+0.75	a	b	r^2	Lower 95%	Upper 95%
	δ	Δδ	Δδ	Δδ	Δδ					
1-OCH_3	3.98	0.60	1.7	3.91	6.92	10.0271	–7910	0.9934	7.5463	12.5079
2-H	6.25	0.30	0.86	1.96	3.45	4.9939	–0.3863	0.9940	3.8115	6.1763
3-OCH_3	3.89	0.06	0.20	0.45	0.76	1.1078	–0.0825	0.9977	0.9474	1.2682
4-H	6.37	0.09	0.34	0.74	1.27	1.8549	–0.1436	0.9965	1.5182	2.1917
N-CH_3	3.97	0	0.05	0.40	0.71	1.1878	–0.1925	0.9847	0.7379	1.6377
5-OCH_3	3.76	0.02	0.14	0.34	0.73	1.1078	–0.1425	0.9744	0.5611	1.6545
6-OCH_3	3.92	0.03	0.06	0.08	0.11	0.1193	0.0215	0.9651	0.0503	0.1884
7-H	6.88	–0.03	0.03	0.08	0.19	0.3322	–0.0675	0.9754	0.1718	0.4926
8-H	8.14	0.06	0.25	0.59	1.08	1.6081	–0.1583	0.9925	1.1837	2.0325

a = slope of the regression line. b = intercept. r^2 = coefficient of determination. Lower 95% confidence interval. Upper 95% confidence interval

Table S4. Chemical shifts (δ, ppm) and chemical shifts difference (Δδ, ppm) of compound 4 under different mole ratios of Eu(dpm)3 to the substrate.

ratio / gp	+0	+0.125	+0.25	+0.5	+0.75	a	b	r^2	Lower 95%	Upper 95%
	δ	Δδ	Δδ	Δδ	Δδ					
1-OCH_3	3.92	0.84	1.76	4.36	6.75	9.6231	–0.4819	0.9980	8.3219	10.9242
2-H	6.27	0.44	0.95	2.23	3.46	4.8868	–0.2153	0.9990	4.4270	5.3466
NCH_3	3.79	0.08	0.17	0.40	0.61	0.8597	–0.0342	0.9989	0.7747	0.9446
5-H	7.28	0.09	0.16	0.40	0.60	0.8393	–0.0285	0.9956	0.6694	1.0092
6-H	7.56	0.05	0.07	0.14	0.24	0.3064	0.0005	0.9793	0.1709	0.4420
7-H	7.17	0.03	0.04	0.10	0.15	0.2007	–0.0015	0.9874	0.1316	0.2697
8-H	8.32	0.17	0.32	0.76	1.16	1.6149	–0.5360	0.9976	1.3758	1.8540
1'-H	6.49	0.14	0.27	0.63	0.97	1.3492	–0.0456	0.9982	1.1730	1.5253
2'-H	5.46	0.09	0.17	0.37	0.57	0.7756	–0.0151	0.9988	0.6948	0.8564
3'-H	1.56	0.04	0.08	0.24	0.35	0.5166	–0.0324	0.9909	0.3658	0.6674

a = slope of the regression line. b = intercept. r^2 = coefficient of determination. Lower 95% confidence interval. Upper 95% confidence interval

Table S5. Chemical shifts (δ, ppm) and chemical shifts difference (Δδ, ppm) of compound 5 under different mole ratios of Eu(dpm)3 to the substrate.

ratio / gp	+0	+0.125	+0.25	+0.5	+0.75	a	b	r^2	Lower 95%	Upper 95%
	δ	Δδ	Δδ	Δδ	Δδ					
1-OCH_3	3.97	0.58	1.86	3.80	6.03	8.5708	–0.4144	0.9983	7.4996	9.6421
2-H	6.29	0.30	0.95	1.96	3.09	4.3959	–0.2108	0.9986	3.9021	4.8897
NCH_3	3.59	0.06	0.19	0.38	0.57	0.8027	–0.0261	0.9967	0.6612	0.9442
5-OCH_3	3.94	0.06	0.15	0.27	0.40	0.5315	0.0041	0.9956	0.4239	0.6391
6-H	7.07	0	0.05	0.19	0.33	0.5356	–0.0751	0.9975	0.4548	0.6164
7-H	7.18	0	0.01	0.05	0.10	0.1627	–0.0261	0.9841	0.0999	0.2255
8-H	7.88	0.10	0.37	0.77	1.20	1.7302	–0.0929	0.9978	1.4851	1.9752
1'-H	6.66	0.08	0.29	0.59	0.94	1.3478	–0.0725	0.9975	1.1434	1.5522
2'-H	5.54	0.04	0.17	0.36	0.54	0.7878	–0.0425	0.9955	0.6274	0.9482
3'-CH_3	1.53	0.04	0.10	0.20	0.30	0.4122	–0.0075	0.9990	0.3718	0.4526

a = slope of the regression line. b = intercept. r^2 = coefficient of determination. Lower 95% confidence interval. Upper 95% confidence interval

Table S6. Chemical shifts (δ, ppm) and chemical shifts difference (Δδ, ppm) of compound 6 under different mole ratios of Eu(dpm)3 to the substrate.

ratio / gp	+0	+0.125	+0.25	+0.5	+0.75	a	b	r^2	Lower 95%	Upper 95%
	δ	Δδ	Δδ	Δδ	Δδ					
1-OCH_3	3.95	0.80	1.88	3.93	6.19	8.5803	–0.2858	0.9995	8.0214	9.1393
2-H	6.28	0.41	0.92	2.01	3.15	4.3919	–0.1617	0.9996	4.1317	4.6520
NCH_3	3.62	0.06	0.12	0.34	0.53	0.7742	–0.052	0.9948	0.6039	0.9445
5-OCH_3	3.93	0.04	0.13	0.39	0.71	1.0807	–0.1215	0.9915	0.7755	1.3858
6-OCH_3	3.89	0.05	0.05	0.11	0.13	0.1437	0.0266	0.9335	0.0270	0.2604
7-H	6.88	0.01	0.01	0.10	0.16	0.2603	–0.0358	0.9642	0.1078	0.4129
8-H	8.01	0.14	0.31	0.74	1.15	1.6353	–0.0793	0.9990	1.4783	1.7922
1'-H	6.59	0.12	0.25	0.59	0.92	1.2963	–0.0566	0.9986	1.1491	1.4434
2'-H	5.55	0.06	0.12	0.34	0.52	0.7593	–0.0485	0.9946	0.5894	0.9292
3'-CH3	1.53	0.03	0.05	0.19	0.29	0.4393	–0.0385	0.9841	0.2694	0.6092

a = slope of the regression line. b = intercept. r^2 = coefficient of determination. Lower 95% confidence interval. Upper 95% confidence interval
